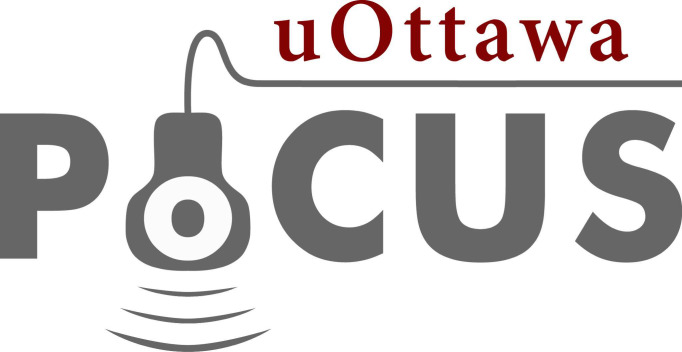# Comparison of Clerkship Directors’ Expectations of Physical Examination Skills with Point-of-care Ultrasound Skills Using the RIME Framework

**DOI:** 10.24908/pocus.v6i2.15192

**Published:** 2021-11-23

**Authors:** Valérie Desjardins, Paul Pageau, Barbara Power, Isabelle Burnier, Carolina Souza, Warren J Cheung, Michael Y. Woo

**Affiliations:** 1 University of Ottawa; 2 Department of Emergency Medicine, University of Ottawa and The Ottawa Hospital; 3 Department of Medicine, University of Ottawa and The Ottawa Hospital; 4 Department of Radiology, University of Ottawa and The Ottawa Hospital; 5 Department of Emergency Medicine, University of Ottawa and Ottawa Hospital Research Institute

**Keywords:** point-of-care ultrasound, undergraduate clerkship

## Abstract

**Background:** The expectations of point-of-care ultrasound (PoCUS) in undergraduate clerkship at the University of Ottawa has not been described. We compared clerkship directors’ expectations of physical examination skills with PoCUS skills, before and after completing the clerkship rotation. **Methods: ** A pilot-tested, expert developed, bilingual on-line survey consisting of 15 questions was sent to all clerkship directors (23) in December 2019. The survey included questions regarding the expectations of medical students with respect to physical examination and PoCUS using the RIME Framework: none, reporter, interpreter, manager, educator. **Results:** The response rate was 60.9% (14/23). With regards to physical exam skills, 82.8% of directors had no expectations or expected students to be reporters when starting clerkship. At graduation, 77.5% of directors expected students to be interpreters, managers, or educators. For PoCUS, 100.0% of directors had no expectations or expected students to be reporters when starting clerkship. At clerkship completion, 33.0% of directors felt that students should be interpreters or managers for PoCUS skills. **Conclusions: **Clerkship directors have low expectations of PoCUS skills for entering and graduating clerks when compared with their physical examination skills despite formal pre-clerkship PoCUS objectives. Enhanced communication and targeted education of directors could improve the PoCUS curriculum.

## Background

Point-of-care ultrasound (PoCUS), a diagnostic or procedural ultrasound performed by a physician at the bedside, is changing the way many clinicians practice medicine. PoCUS is described as being a useful adjunct to the physical examination, such as the stethoscope or reflex hammer, that can improve clinical judgement and patient satisfaction 1, 2, 3, 4, 5. In 2016, a survey indicated that approximately half of Canadian medical schools teach PoCUS to their medical students 6. The University of Ottawa has physical examination skills objectives throughout the four years of medical school, and more recently, PoCUS skills objectives for their pre-clerkship students (https://med.uottawa.ca/undergraduate/students/student-zone/pre-clerkship).

The current expectations regarding PoCUS skills in undergraduate clerkship at the University of Ottawa medical school has not been described. This survey compares the expectations of PoCUS examination to the physical exam. PoCUS skills were taught in conjunction with Physicians Skills Development (PSD). PoCUS skills have also been described as an adjunct to the physical exam. The objective of the survey is to compare the Clerkship Directors’ expectations of overall student performance before and after completing clerkship as a whole, with respect to physical examination skills and PoCUS skills using the RIME framework 7. 

The framework used is based on the RIME mnemonic: Reporter, Interpreter, Manager, Educator. Anchor definitions were applied in the following manner: a reporter can obtain and communicate the examination (eg. inspect, palpate, percuss, auscultate or identify PoCUS abnormalities), an interpreter can analyze and interpret the examination (eg. find possible cause of abnormality), a manager can integrate the examination and propose treatments (eg. determine management plan), and an educator can teach other students how to perform and integrate the findings of an examination (eg. teach pre-clerkship students to be a reporter, interpreter, and manager of a particular exam).

## Methods

A bilingual (English/French) online survey was administered in December 2019. The survey was developed by a PoCUS expert from Emergency Medicine, a radiologist, the anglophone and francophone directors of Clinical Skills at the University of Ottawa medical school, and a second-year medical student. The survey was pilot tested with three physicians (Family Medicine and Emergency Medicine) and six medical students. The Ottawa Health Science Network Research Ethics Board (OHSN-REB) waived its review and the project was deemed REB exempt. 

The final survey was composed of three sections with a total of 15 questions: general demographics, expectations for the physical examination, expectation for the PoCUS examination. Five types of examinations were surveyed for both the physical examination and PoCUS: cardiovascular, respiratory, abdominal, musculoskeletal, and thyroid (online supplementary Appendix A). Respondents were asked to categorize each physical examination skill or PoCUS item based on anchors from the RIME Framework that described their expectation of the specific examination for the medical student, pre and post clerkship 7. Differences between the five types of examinations were assessed by an unpaired t-test. The five types of examinations were then grouped together to compare the expectations for the physical examination and the POCUS examinations directly.

The survey was distributed by SurveyMonkey (San Mateo, USA) on December 3, 2019 to a total of 23 clerkship rotation directors. Follow-up reminders were sent to non-responders after one week and two weeks.

A single data abstractor collected and analyzed the data using GraphPad Prism (San Diego, USA). Descriptive statistics were performed. Responses were kept anonymous, and all data was reported in aggregate. 

## Results

14 of the 23 undergraduate clerkship directors at the University of Ottawa medical school responded to the survey, for a response rate of 60.9%. The demographic information and PoCUS use of respondents are summarized in Table 1. The majority of clerkship directors (57.1%) have been practicing medicine for over 10 years and come from various specialties. Half of the respondents use PoCUS in their medical practice. Of the seven respondents who do not currently use POCUS, three would practice if given the opportunity to learn (21.4%), two would consider it (14.3%), and two would not practice PoCUS even if given the opportunity (14.3%). There was no statistical difference between the five types of examinations. 

**Table 1:** Demographics of clerkship rotation directors.

**Years as practicing physician**	**N (%)**
<5 years	3 (21.4)
5-10 years	3 (21.4)
>10 years	8 (57.1)
**Physician specialties**	
Family Medicine	2 (14.3)
Obstetrics and Gynecology	2 (14.3)
Anesthesiology	2 (14.3)
Psychiatry	1 (7.1)
General Internal Medicine	2 (14.3)
Geriatrics	1 (7.1)
Endocrinology	1 (7.1)
Emergency Medicine	1 (7.1)
Pediatrics	1 (7.1)
Otolaryngology	1 (7.1)
**PoCUS use in medical practice**	
Yes	7 (50.0)
No	7 (50.0)
N=14; PoCUS – point-of-care ultrasound	

### Expectations upon entering clerkship

Regarding the physical examination, most responders (82.8 %) had no expectations (30.8%) or expected students to be reporters (52.0%). Regarding the PoCUS examination for students entering clerkship, all respondents had no expectations (66.8%) or expected students to be reporters (33.2%).

### Expectations upon completing clerkship

Regarding the physical examination, 77.5% of the clerkship directors felt the student should be interpreters (38.3%), managers (27.6%), or educators (11.6%) of specific physical examinations, while the remaining respondents (22.5%) had no expectations (8.7%) or expected the graduating students to be reporters (13.8%). For students completing clerkship, 33.0% of respondents believe students should be interpreters (18.4%) or managers (14.8%). 

### Expectations of clerkship directors who use PoCUS

Clerkship directors who use PoCUS in their practice had higher ultrasound expectations for students completing clerkship than those who do not use PoCUS. Differences between clerkship directors who use PoCUS and those who do not were assessed by an unpaired t-test followed by a Holm-Sidak post-hoc test, with the data for the five types of examinations grouped to compare the RIME framework. 40.0% of clerkship directors who use PoCUS in their practice expect the graduating students to be interpreters or managers, compared to 26.4% of non-PoCUS users (p<0.05).

Clerkship director’s expectation of students starting and completing clerkship for the physical exam and PoCUS are illustrated in Table 2.

**Table 2:** Expectations of clerkship directors entering and leaving clerkship for physical exam andPoCUS using the RIME Framework.

	**Physical Examination Skills Expectations**		**PoCUS Skills Expectations**	
	**Entering Clerkship**	**Leaving Clerkship**	**Entering Clerkship**	**Leaving Clerkship**
	**N (%)**	**N (%)**	**N (%)**	**N (%)**
None	67 (30.8)	23 (8.7)	141 (66.8)	110 (51.8)
Reporter	125 (52.0)	29 (13.8)	69 (33.2)	31 (15.0)
Interpreter	39 (17.2)	88 (38.3)	0	36 (18.4)
Manager	0	65 (27.6)	0	33 (14.8)
Educator	0	26 (11.6)	0	0
N represents the amount of times the clerkship directors have selected the RIME Framework description for the five types of examinations surveyed (cardiovascular, respiratory, abdominal, musculoskeletal, and thyroid).				

## Discussion

The physical examination skills objectives are clearly stated in the undergraduate medical education curriculum at the University of Ottawa, for both the pre-clerkship and clerkship programs. There are clear and available PoCUS objectives for pre-clerkship. However, clerkship PoCUS objectives are not easily available for all members of the Undergraduate Medical Education Faculty, since the objectives on the clerkship website are split into the different rotations and none of them explicitly mention PoCUS. Using the RIME framework, the results of this study indicate that the expectations for the physical examination increase for students completing clerkship. It is surprising that only 17.2% of clerkship directors felt that a student entering clerkship should be able to interpret a physical examination, despite objectives in pre-clerkship describing being able to analyze and interpret the abnormalities of a physical examination. Similarly, Wenrich et al. found that preclinical faculty and medical students had much higher expectations than clerkship faculty for most clinical skills 8. 

PoCUS is described as being an useful adjunct to the physical examination that improves clinical judgement and patient satisfaction 1, 2, 3, 4, 5. Formal pre-clerkship PoCUS curriculum objectives were introduced in 2018 at the University of Ottawa. Nonetheless, clerkship directors have little to no expectations with respect PoCUS skills for the student entering clerkship (66.8% had no expectations and 33.2% expected reporters). There is a marginal increase in these expectations for the graduating clerk, with 33.0% of directors expecting interpreters or managers of PoCUS skills. Overall, this study highlights the need to strengthen communication of expectations between the pre-clerkship and clerkship faculty and to promote familiarity with the pre-clerkship PoCUS skills objectives, as clerkship directors are perhaps not aware of any PoCUS teaching in pre-clerkship or that objectives were added to the curriculum. It is important to establish formal clerkship objectives to provide continued PoCUS learning for medical students at the University of Ottawa. This would greatly benefit medical students by providing consistent communication of what skills and abilities are expected of them, especially when entering clerkship 8, 9, 10. It is also possible that clerkship directors feel that PoCUS is not a useful clinical skill for the graduating medical student to possess, despite evidence that ultrasonography is a valuable teaching tool that enhances medical education 9. Since the clerkship directors who do not use PoCUS in their medical practice tend to have lower expectations of their clerks in regard to PoCUS skills, targeted education of these directors could be an important step in the implementation of PoCUS objectives in clerkship.

As with all survey designs, one limitation of this study is the possibility of sample bias. Program directors using PoCUS in their medical practice might have been more likely to respond to the survey than those who do not. Additionally, one clerkship director misunderstood the purpose of the study as described in the survey, and indicated their expectations in relation to the completion of their specific rotation, rather than completion of clerkship as a whole. Finally, it’s possible that respondents were not familiar with the RIME framework used to the define level of performance of their students. However, we attempted to mitigate this by providing descriptive anchors for each level of the framework.

## Conclusion

Clerkship rotation directors have little expectations of entering and graduating clerks with regards to their PoCUS skills when compared with their physical examination skills. These differing expectations exist despite formal PoCUS objective in the pre-clerkship curriculum. Improved communication between pre-clerkship and clerkship faculty as well as targeted education of clerkship directors could be an important step to improve the implementation of a PoCUS curriculum in clerkship. Further study is required to uncover the cause of this discrepancy between physical examination skills and POCUS skills expectations.

## Conflicts of Interest Notification

Valérie D. Desjardins, Dr. Paul Pageau, Dr Barbara Power, Dr Isabelle Burnier, and Dr. Warren J. Cheung have no conflicts of interest. Dr Carolina Souza - Advisory board: AstraZeneca, Boehringer-Ingelheim. Consultant fees and honorarium: Pfizer, Boehringer-Ingelheim, AstraZeneca, Hoffmann-La Roche. Educational Grant: Boehringer-Ingelheim. Dr Michael Y. Woo, MD – Teaching Chair - POCUS, Undergraduate Medical Education, University of Ottawa. 

## Funding:

There was no funding for this program evaluation.

## Supplementary Material 

Supplemental Appendix A English Survey

Supplemental Appendix B French Survey

Figure S1U of Ottawa POCUS logo