# Supplementary material for: Comparison of Clerkship Directors’ Expectations of Physical Examination Skills with Point-of-care Ultrasound Skills Using the RIME Framework
Source: POCUS J. 2021 Nov 23;6(2):93–6. doi: 10.24908/pocus.v6i2.15192 (PMC9979901; doi:10.24908/pocus.v6i2.15192)
Supplement: Supplemental Appendix A [file pocusj-06-15192-s001.pdf]

Point-of-Care Ultrasound Training

General Information

Thank you for taking the time to complete this needs assessment regarding the role of Point-of-care Ultrasound in the clerkship program at the University of Ottawa.

Point-of-Care Ultrasound (POCUS) is being integrated into medical school curricula across Canada. POCUS is the performance of ultrasound by the clinician at the bedside that is limited in scope, and problem oriented.

The goal of this survey is to compare your expectations on POCUS training to physical examination training in your program.

* 1. What is your specialty as a doctor?

[ ]

* 2. How many years have you been an attending physician?

- ( ) <5 years
- ( ) 5-10 years
- ( ) >10 years

* 3. Do you use Point-of-Care Ultrasound (PoCUS) in your practice?

- ( ) Yes
- ( ) No

Comment:

[ ]

uOttawa POCUS

Point-of-Care Ultrasound Training

General Information

* 4. Would you practice PoCUS given the opportunity?

- ○ Yes
- ○ No
- ○ Maybe

Comment:

uOttawa POCUS

Point-of-Care Ultrasound Training

Physical Examination

Using the RIME Framework. Reporter, Interpreter, Manager, Educator, please indicate your expectations of medical student entering clerkship compared with at the end of clerkship with respect to Physical Examination skills.
<ref: Pangaro, L. (1999). A new vocabulary and other innovations for improving descriptive in-training evaluations. Academic Medicine, 74(11), 1203-7.>

Reporter of a physical exam: Able to obtain and communicate the physical exam (eg. inspect, palpate, percuss, auscultate)
Interpreter of a physical exam: Able to analyze and interpret the physical exam (eg. find possible cause of abnormality)
Manager of a physical exam: Able to integrate the physical exam and propose treatments (eg. determine management plan)
Educator of a physical exam: Able to explain to the patient and to teach students how to perform and integrate the findings of the physical exam (eg. teach preclerkship students to be a reporter, interpreter, and manager of a physical exam)

* 5. For these abnormal findings in the *cardio-vascular* **physical exam**, I expect students to be a… (please select your answer using the RIME framework)

	**Entering** Clerkship	**Leaving** Clerkship
Valve dysfunction		
Acute coronary syndrome		
Cardiomyopathy		
Pericardial effusion		
Peripheral vascular disease		

Comment:

* 6. For these abnormal findings in the *respiratory* **physical exam**, I expect students to be a… (please select your answer using the RIME framework)

	**Entering** clerkship	**Leaving** clerkship
Pneumonia		
Pleural effusion		
Pneumothorax		

Comment:

* 7. For these abnormal findings in the *abdominal* **physical exam**, I expect students to be a… (please select your answer using the RIME framework)

	**Entering** clerkship	**Leaving** clerkship
Appendicitis		
Peritonitis in trauma		
Cholecystitis		
Chronic liver disease		
Acute kidney failure		
Abdominal aortic aneurysm		

Comment:

* 8. For these abnormal findings in the *musculoskeletal* **physical exam**, I expect students to be a… (please select your answer using the RIME framework)

	**Entering** clerkship	**Leaving** clerkship
Joint effusion		
Muscle/Tendon rupture		
Joint dislocation		
Superficial Abscess		
Cellulitis		

Comment:

* 9. For these abnormal findings in the *thyroid and lymph node* **physical exam**, I expect students to be a… (please select your answer using the RIME framework)

	**Entering** clerkship	**Leaving** clerkship
Thyroid nodule		
Lymph node abnormalities		

Comment:

Point-of-Care Ultrasound Training

Point-of-Care Ultrasound (PoCUS)

Using the RIME Framework. Reporter, Interpreter, Manager, Educator, please indicate your expectations of medical student entering clerkship compared with at the end of clerkship with respect to PoCUS skills.
<ref: Pangaro, L. (1999). A new vocabulary and other innovations for improving descriptive in-training evaluations. Academic Medicine, 74(11), 1203-7.>

Reporter of a PoCUS exam: Able to obtain and communicate these PoCUS findings (eg. identify PoCUS anatomy and abnormalities)
Interpreter of a PoCUS exam: Able to analyze and interpret these PoCUS findings (eg. find possible causes of PoCUS abnormalities)
Manager of a PoCUS exam: Able to integrate these PoCUS findings and propose treatments (eg. determine management plan)
Educator of a PoCUS exam: Able to explain to the patient and to teach students how to perform and integrate the findings of a PoCUS scan (eg. teach preclerkship students to be a reporter, interpreter, and manager of a PoCUS exam)

* 10. For these abnormal findings in the *cardio-vascular* **PoCUS exam**, I expect students to be a… (please select your answer using the RIME framework)

	**Entering** clerkship	**Leaving** clerkship
Valve dysfunction		
Acute coronary syndrome		
Cardiomyopathy		
Pericardial effusion		
Peripheral vascular disease		

Comment:

* 11. For these abnormal findings in the *respiratory* **PoCUS exam**, I expect students to be a… (please select your answer using the RIME framework)

	**Entering** clerkship	**Leaving** clerkship
Pneumonia		
Pleural effusion		
Pneumothorax		

Comment:

* 12. For these abnormal findings in the *abdominal* **PoCUS exam**, I expect students to be a… (please select your answer using the RIME framework)

	**Entering** clerkship	**Leaving** clerkship
Appendicitis		
Peritonitis in trauma		
Cholecystitis		
Chronic liver disease		
Acute renal disease		
Abdominal aortic aneurysm		

Comment:

* 13. For these abnormal findings in the *musculoskeletal* **PoCUS exam**, I expect students to be a… (please select your answer using the RIME framework)

	**Entering** clerkship	**Leaving** clerkship
Joint effusion		
Muscle/Tendon rupture		
Joint dislocation		
Superficial Abscess		
Cellulitis		

Comment:

* 14. For these abnormal findings in the *thyroid and lymph node* **PoCUS exam**, I expect students to be a… (please select your answer using the RIME framework)

	**Entering** clerkship	**Leaving** clerkship
Thyroid nodule		
Lymph node abnormalities		

Comment:

* 15. Do you have defined objectives and a curriculum for PoCUS in your clerkship rotation?

- Yes
- No

Comments?

uOttawa POCUS

Point-of-Care Ultrasound Training

16. Any comments?
