# Supplementary material for: Comparison of Clerkship Directors’ Expectations of Physical Examination Skills with Point-of-care Ultrasound Skills Using the RIME Framework
Source: POCUS J. 2021 Nov 23;6(2):93–6. doi: 10.24908/pocus.v6i2.15192 (PMC9979901; doi:10.24908/pocus.v6i2.15192)
Supplement: Supplemental Appendix B [file pocusj-06-15192-s002.pdf]

uOttawa POCUS

Formation en échographie au point d'intervention

Renseignements généraux

Merci de prendre le temps de répondre à ce sondage sur le rôle de l'échographie au point d'intervention dans le programme d'externat à l'Université d'Ottawa.

L'échographie au point d'intervention (POCUS) est en voie d'intégration dans les programmes d'études en médecine au Canada. POCUS est la réalisation d'une échographie par le clinicien au chevet du patient, dont les objectifs sont limités et qui sont axés sur les problèmes.

Le but de ce sondage est de comparer vos attentes à l'égard de la formation POCUS à celle de l'examen physique dans votre programme.

* 1. Quelle est votre spécialité en tant que médecin ?

* 2. Depuis combien d'années êtes-vous médecin traitant ?

- <5 ans
- 5-10 ans
- >10 ans

* 3. Utilisez-vous l'échographie au point d'intervention (PoCUS) dans votre pratique ?

- Oui
- Non

Commentaire:

Formation en échographie au point d'intervention

Renseignement généraux

* 4. Pratiqueriez-vous PoCUS si l'occasion se présentait ?

- Oui
- Non
- Peut-être

Commentaire:

Formation en échographie au point d'intervention

Examen physique

En utilisant le cadre RIME, Rapporteur, Interprète, Manager, Éducateur, veuillez indiquer vos attentes à l'égard des étudiants en médecine qui débutent un stage par rapport aux étudiants qui terminent leur stage en ce qui concerne les compétences pour les examens physiques.
<ref: Pangaro, L. (1999). A new vocabulary and other innovations for improving descriptive in-training evaluations. Academic Medicine, 74(11), 1203-7.>

Rapporteur d'un examen physique : Capable de faire et de communiquer l'examen physique (p. ex. inspecter, palper, percuter, ausculter)
Interprète d'un examen physique : Capable d'analyser et d'interpréter l'examen physique (p. ex. trouver la cause possible de l'anomalie)
Manager d'un examen physique : Capable de proposer des traitements (p. ex. déterminer le plan de gestion)
Éducateur d'un examen physique : Capable d'expliquer au patient et d'enseigner aux étudiants comment effectuer et comprendre les résultats de l'examen physique (par exemple, enseigner aux étudiants à effectuer un examen physique).

* 5. Pour ces résultats anormaux à l'**examen physique** *cardio-vasculaire*, je m'attends à ce que les étudiants soient... (veuillez choisir votre réponse en utilisant le cadre RIME)

	**Entrant** l'externat	**Quittant** l'externat
Valvulopathies		
Syndrome coronarien aigu		
Cardiomyopathie		
Épanchement péricardique		
Maladie vasculaire périphérique		

Commentaires:

* 6. Pour ces résultats anormaux à **l'examen physique** du *thorax*, je m'attends à ce que les étudiants soient... (veuillez choisir votre réponse en utilisant le cadre RIME)

	**Entrant** l'externat	**Quittant** l'externat
Pneumonie		
Épanchement pleural		
Pneumothorax		

Commentaires:

* 7. Pour ces résultats anormaux à **l'examen physique** de *l'abdomen*, je m'attends à ce que les étudiants soient... (veuillez choisir votre réponse en utilisant le cadre RIME)

	**Entrant** l'externat	**Quittant** l'externat
Appendicite		
Péritonite		
Cholécystite		
Maladie hépatique chronique		
Insuffisance rénale aigue		
Anévrisme de l'aorte abdominale		

Commentaires:

* 8. Pour ces résultats anormaux à **l'examen physique** *MSK*, je m'attends à ce que les étudiants soient... (veuillez choisir votre réponse en utilisant le cadre RIME)

	**Entrant** l'externat	**Quittant** l'externat
Hydarthrose		
Rupture du muscle/tendon		
Luxation articulaire		
Abcès superficiel		
Cellulite		

Commentaires:

* 9. Pour ces résultats anormaux à l'**examen physique** de la *thyroïde et des ganglions lymphatiques*, je m'attends à ce que les étudiants soient... (veuillez choisir votre réponse en utilisant le cadre RIME)

	**Entrant** l'externat	**Quittant** l'externat
Nodule thyroïdien		
Anomalies des ganglions lymphatiques		

Commentaires:

Formation en échographie au point d'intervention

Échographie au point d'intervention (PoCUS)

En utilisant le cadre RIME, Rapporteur, Interprète, Manager, Éducateur, veuillez indiquer vos attentes à l'égard des étudiants en médecine qui débutent un stage par rapport aux étudiants qui terminent leur stage en ce qui concerne les compétences pour l'échographie au point d'intervention (PoCUS).
<ref: Pangaro, L. (1999). A new vocabulary and other innovations for improving descriptive in-training evaluations. Academic Medicine, 74(11), 1203-7.>

Rapporteur de PoCUS : Capable de faire et de communiquer l'image PoCUS (p. ex. identifier l'anatomie et les anomalies sur l'image PoCUS)
Interprète de PoCUS : Capable d'analyser et d'interpréter l'image PoCUS (p. ex. trouver la cause possible de l'anomalie)
Manager de PoCUS : Capable d'interpréter l'image et proposer des traitements (p. ex. déterminer le plan de gestion)
Éducateur de PoCUS : Capable d'expliquer au patient et d'enseigner aux étudiants comment effectuer et comprendre les résultats de PoCUS (par exemple, enseigner aux étudiants à effectuer un image PoCUS).

* 10. Pour ces résultats anormaux à l’image **PoCUS** *cardio-vasculaire*, je m'attends à ce que les étudiants soient... (veuillez choisir votre réponse en utilisant le cadre RIME)

	**Entrant** l'externat	**Quittant** l'externat
Valvulopathies		
Syndrome coronarien aigu		
Cardiomyopathie		
Épanchement péricardique		
Maladie vasculaire périphérique		

Commentaires:

* 11. Pour ces résultats anormaux à l’image **PoCUS** du *thorax*, je m'attends à ce que les étudiants soient... (veuillez choisir votre réponse en utilisant le cadre RIME)

	**Entrant** l'externat	**Quittant** l'externat
Pneumonie		
Épanchement pleural		
Pneumothorax		

Commentaires:

* 12. Pour ces résultats anormaux à l’image **PoCUS** de *l'abdomen*, je m'attends à ce que les étudiants soient... (veuillez choisir votre réponse en utilisant le cadre RIME)

	**Entrant** l'externat	**Quittant** l'externat
Appendicite		
Péritonite		
Cholécystite		
Maladie hépatique chronique		
Insuffisance rénale aigue		
Anévrisme de l'aorte abdominale		

Commentaires:

* 13. Pour ces résultats anormaux à l’image **PoCUS** MSK, je m'attends à ce que les étudiants soient... (veuillez choisir votre réponse en utilisant le cadre RIME)

	**Entrant** l'externat	**Quittant** l'externat
Hydarthrose		
Rupture du muscle/tendon		
Luxation articulaire		
Abcès superficiel		
Cellulite		

Commentaires:

* 14. Pour ces résultats anormaux à l'examen **PoCUS** de la *thyroïde et des ganglions lymphatiques*, je m'attends à ce que les étudiants soient... (veuillez choisir votre réponse en utilisant le cadre RIME)

	**Entrant** l'externat	**Quittant** l'externat
Nodule thyroïdien		
Anomalies des ganglions lymphatiques		

Commentaires:

* 15. Avez-vous des objectifs et un curriculum clairement définis pour PoCUS dans votre programme d'externat ?

- Oui
- Non

Commentaires:

Formation en échographie au point d'intervention

16. Est-ce que vous avez des commentaires?
